# Global Ocean Particulate Organic Phosphorus, Carbon, Oxygen for Respiration, and Nitrogen (GO-POPCORN)

**DOI:** 10.1038/s41597-022-01809-1

**Published:** 2022-11-11

**Authors:** Tatsuro Tanioka, Alyse A. Larkin, Allison R. Moreno, Melissa L. Brock, Adam J. Fagan, Catherine A. Garcia, Nathan S. Garcia, Skylar D. Gerace, Jenna A. Lee, Michael W. Lomas, Adam C. Martiny

**Affiliations:** 1 Department of Earth System Science, University of California, Irvine, Irvine, CA 92697 USA; 2 Department of Ecology and Evolutionary Biology, University of California, Irvine, Irvine, CA 92697 USA; 3 Atmospheric & Oceanic Sciences, University of California, Los Angeles, Los Angeles, CA 90095 USA; 4 Center for Microbial Oceanography: Research and Education (C-MORE), University of Hawaii at Manoa, Honolulu, HI 96822 USA; 5 Department of Geosciences, Princeton University, Princeton, NJ 08544 USA; 6 Bigelow Laboratory for Ocean Sciences, East Boothbay, ME 04544 USA

**Keywords:** Marine chemistry, Element cycles

## Abstract

Concentrations and elemental stoichiometry of suspended particulate organic carbon, nitrogen, phosphorus, and oxygen demand for respiration (C:N:P:−O_2_) play a vital role in characterizing and quantifying marine elemental cycles. Here, we present Version 2 of the Global Ocean Particulate Organic Phosphorus, Carbon, Oxygen for Respiration, and Nitrogen (GO-POPCORN) dataset. Version 1 is a previously published dataset of particulate organic matter from 70 different studies between 1971 and 2010, while Version 2 is comprised of data collected from recent cruises between 2011 and 2020. The combined GO-POPCORN dataset contains 2673 paired surface POC/N/P measurements from 70°S to 73°N across all major ocean basins at high spatial resolution. Version 2 also includes 965 measurements of oxygen demand for organic carbon respiration. This new dataset can help validate and calibrate the next generation of global ocean biogeochemical models with flexible elemental stoichiometry. We expect that incorporating variable C:N:P:-O_2_ into models will help improve our estimates of key ocean biogeochemical fluxes such as carbon export, nitrogen fixation, and organic matter remineralization.

## Abstract (summary)

**Table:** 

Measurement(s)	particulate matter • particulate carbon oxygen demand • particulate phosphorus
Technology Type(s)	elemental analyzer • potassium dichromate • ash-hydrolysis
Factor Type(s)	location • period
Sample Characteristic - Environment	ocean
Sample Characteristic - Location	global

## Background & Summary

The elemental ratio between carbon (C), nitrogen (N), phosphorus (P), and oxygen (O_2_) demand for respiration is a fundamental quantity that couples nutrient uptake by primary producers, organic carbon export, and remineralization^1–3^. Most ocean biogeochemical models from the pre-CMIP6 era have exclusively used the fixed canonical Redfield C:N:P and respiration quotient -O_2_:C of 106:16:1 and 1, respectively, to link nutrient uptake and convert to and from organic carbon. However, it is now widely accepted in the oceanographic community that C:N:P:-O_2_ in the surface ocean are variable through space and time. Previous global compilation studies^4,5^ have shown that C:P and N:P are systematically higher than the Redfield ratios of 106:1 and 16:1 in the nutrient-deplete subtropical gyres, lower in the nutrient-rich subpolar and polar regions, and approximately equal to the Redfield values in the tropical and upwelling regions. The respiration quotient of particulate organic matter (POM) in terms of -O_2_:C and -O_2_:P has also been shown to be spatially variable through direct observations and inverse modeling^6–8^. In light of these recent observations, our understanding of the oceanic ecosystem elemental stoichiometry has evolved rapidly over the last ten years.

**Fig. 1:**
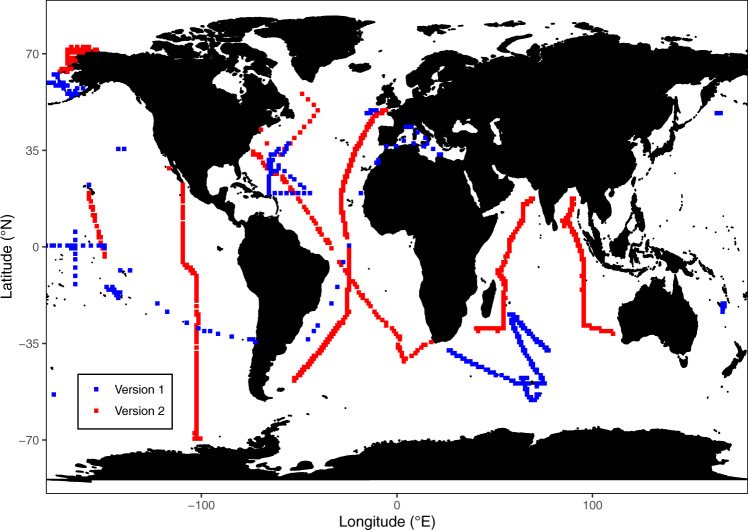
Distribution of paired POC/N/P measurements in the surface ocean. Samples from GO-POPCORN v1 (n = 580) and v2 (n = 2093) are shown in blue and red, respectively.

Here we present Version 2 (“v2”) of the Global Ocean Particulate Organic Phosphorus, Carbon, Oxygen for Respiration, and Nitrogen (GO-POPCORN) dataset (Fig. 1). We refer to Version 1 (“v1”) as a previously published data compilation^9^, in which POC/N/P was collated from 70 cruises and time-series between 1971 and 2010. Version 1 has served multiple purposes, such as calibration and validation of ocean biogeochemical models, including those used in the latest coupled model intercomparison project (CMIP6)^10–12^, and identifying drivers of global-scale spatiotemporal variability in C:N:P^13,14^. However, several limitations of GO-POPCORN v1 were identified. First, there was a significant bias towards regions of frequent oceanographic research, leading to samples being concentrated in the North Atlantic, Eastern North Pacific Ocean, Mediterranean Ocean, and near the Palmer Station in the Southern Ocean (Fig. 1). Second, aggregated data samples were collected using different techniques, such as differing blank measurements and detection limits. Third, a large proportion of measurements came from time-series studies at a fixed geographical location: Hawaiian Ocean Time-series (HOT), Bermuda Atlantic Time-series Study (BATS), and CARIACO Ocean Time-series program.

**Fig. 2:**
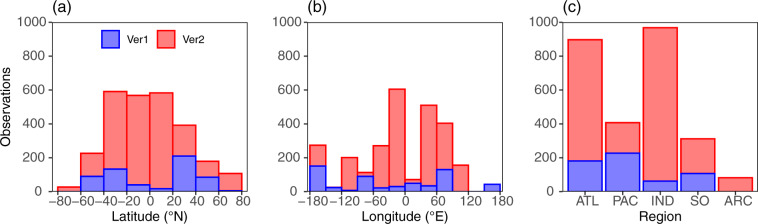
Geographical distribution of paired POC/N/P measurements in the surface ocean. The number of paired POC/N/P measurements binned by (**a**) every 20° of latitude, (**b**) every 30° of longitude, and (**c**) by oceanographic basins for GO-POPCORN v1 (blue) and v2 (red). [Abbreviations: ATL = Atlantic Ocean, PAC = Pacific Ocean, IND = Indian Ocean, SO = Southern Ocean, ARC = Arctic Ocean].

GO-POPCORN v2 is a new compendium of global POC/N/P collected between 2011 and 2020 as part of Bio-GO-SHIP (the Biological initiative for the Global Ocean Ship-based Hydrographic Investigations Program)^15,16^ and the Arctic Integrated Ecosystem Research Program (IERP)^17^. The v2 dataset contains 2581 paired measurements (of which 2093 measurements are from the surface ocean) of POC/N/P and 965 measurements of particulate chemical oxygen demand (PCOD), which is the oxygen needed for full respiration of organic carbon^7^. The new version has a comprehensive geographic range, and the samples were collected across all major oceanic regions from 70°S to 73°N (Fig. 2) across 2188 stations using a consistent methodology and quality control (Table 1).

**Fig. 3:**
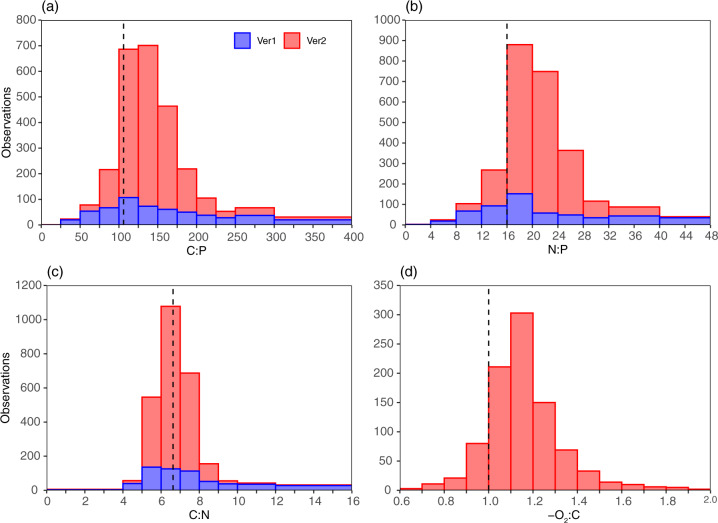
Summary of observed C:N:P:−O_2_ in the surface ocean. The histogram of (**a)** C:P, (**b**) N:P, (**c**) C:N, and (**d**) −O_2_:C from GO-POPCORN v1 (blue) and v2 (red). Black dashed lines are Redfield C:N:P and −O_2_:C of 106:16:1 and 1.0, respectively, for comparison. Please note a difference in the total number of observations for each elemental ratio and that −O_2_:C was not measured in v1.

Median C:N:P for paired surface POM samples from GO-POPCORN v1 and v2 are 140:19:1 and 136:21:1, respectively (Fig. 3). The data spread is noticeably smaller in v2 compared to v1. Specifically, the interquartile range (IQR) in v2 is reduced by a factor of 2–3 compared to that of v1 (IQR of C:P, N:P, C:N in versions 1 and 2 are [103, 13, 2] and [43, 6, 1], respectively). About 90% of observed C:P and N:P from v2 are above the Redfield ratios of 106 and 16, respectively (Fig. 3a,b). This contrasts with v1, where only 75% of samples collected have C:P and N:P above the Redfield ratios. In both versions, the observed mode for C:N is around the Redfield C:N of 6.7, but values are more tightly clustered around 5–8 in v2 (Fig. 3c). The median -O_2_:C from v2 is 1.14, with an IQR of 0.17 (Fig. 3d). Thus, surface organic matter is generally more reduced than pure carbohydrate, with a respiration quotient of 1 (i.e., Redfield -O_2_:C)^18,19^. In summary, both the quantity and the quality of the data have significantly improved in v2 over v1.

## Methods

GO-POPCORN v1 is an exhaustive compilation of POM collected by 70 independent studies and cruises from 1971 to 2010. Refer to the original description paper^9^ for more details on how the v1 dataset was compiled.

GO-POPCORN v2 comprises samples from 12 recent cruises between 2011 and 2020 (Table 1). These sampling efforts have been supported by GO-SHIP (C13.5^20^, I07N^21^, I09N^22^, and P18^23^), SOCCOM and Plymouth Marine Laboratory Atlantic Meridional Transect (AMT-28^24^), National Science Foundation Dimensions of Biodiversity (AE1319^25^, BVAL46^26^, NH1418^27^), and North Pacific Research Board Arctic Integrated Ecosystem Research Program (OS1701^28^, OS1901^28^, SKQ201709S^29^, SKQ201813S^29^).

**Table 1:** Summary of data in GO-POPCORN Version 2, including the number of stations and particulate organic matter (POM) samples and the mean elemental ratios.

Cruise (Program)	Year	#Stations	Latitude		Longitude		POC	PON	POP	PCOD	C:P	N:P	C:N	−O_2_:C	Ref.
			min	max	min	max	(# Samples)				(Geometric mean)				
AE1319 (NSF)	2013	15	32	55	−69	−40	123	111	111	0	145	12	11.6	NA	^25,31,45^
AMT-28 (PML AMT, SOCCOM, NSF)	2018	709	−48	50	−53	−6	741	741	775	771	155	23	6.7	1.2	^8,24,34^
BVAL46 (BATS, NSF)	2011	18	20	39	−66	−64	0	0	197	0	NA	NA	NA	NA	^26,31,45^
C13.5 (GO-SHIP)	2020	112	−41	35	−74	17	112	112	112	0	155	22	7.1	NA	^20^
I07N (GO-SHIP)	2018	719	−30	18	40	69	732	733	727	0	121	19	6.4	NA	^21^
I09N (GO-SHIP)	2016	238	−31	18	85	110	235	235	236	0	134	19	7.1	NA	^22,30,31,34^
NH1418 (NSF)	2014	88	−3	19	−158	−150	159	159	180	0	142	23	6.1	NA	^27,31,33^
P18 (GO-SHIP)	2016–2017	193	−70	29	−116	−100	194	194	194	194	130	21	6.2	1.1	^7,23,32^
OS1701 (Arctic IERP)	2017	30	67	72	−169	−154	106	106	105	0	96	13	7.4	NA	This study
OS1901 (Arctic IERP)	2019	38	63	73	−171	−154	137	137	137	0	150	21	7.2	NA	This study
SKQ201709S (Arctic IERP)	2017	14	63	69	−173	−165	72	72	72	0	142	18	8.0	NA	This study
SKQ201813S (Arctic IERP)	2018	14	63	69	−172	−164	53	53	53	0	113	17	6.7	NA	This study
Summary	2011–2020	2188	−70	73	−173	110	2664	2653	2899	965	137	21	6.7	1.1	

We operationally define the sampling station as a distinct pair of longitude and longitude. Similar descriptions for GO-POPCORN Version 1 are listed in Table 1 of Martiny *et al*.^9^. [Abbreviations: POC = Particulate Organic Carbon, PON = Particulate Organic Nitrogen, POP = Particulate Organic Phosphorus, PCOD = Particulate Chemical Oxygen Demand, BATS = Bermuda Atlantic Time-series Study, GO-SHIP = Global Ocean Ship-based Hydrographic Investigations Program, NSF = National Science Foundation, PML AMT = Plymouth Marine Laboratory Atlantic Meridional Transect, SOCCOM = Southern Ocean Carbon and Climate Observations and Modeling project, IERP = Integrated Ecosystem Research Program].

The POM samples were collected and analyzed using the consistent sampling method described previously^30–33^. Briefly, 3–8 L seawater was collected from the flow-through underway system or CTD. Samples from underway systems were filtered using 30 µm nylon mesh to remove large particles from the sample. Samples were then collected on GF/F filters (Whatman, nominal pore size 0.7 µm) that were precombusted at 500 °C for 5 h to remove any traces of inorganic carbon as well as organic contaminants. Whenever possible, POC, PON, and POP were sampled in triplicate, and PCOD was sampled in sextuplicate. Triplicate sampling occurred hourly in cruises AMT-28 and I07N; every 4 hours for C13.5, I09N, and P18; and once a day for AE1319, BVAL46, NH1418, OS1701, OS1901, SKQ201709S, and SKQ201813S. Differences in the sample collection are based on differences in the hypotheses being tested. For example, hourly sampling in AMT-28 and I07N is aimed toward capturing the diurnal changes in elemental stoichiometry^34^.

POC and PON samples were measured using a CN Flash 1112 EA or 240-XA/440-XA elemental analyzer and were calibrated using a known quantity of atropine (C_17_H_23_NO_3_). Inorganic carbonates were removed using concentrated hydrochloric acid fumes before analysis by storing filters in a desiccator for 24 hours. The mean detection limits for POC and PON, defined as ~3x standard deviation of the low standards, are ~2.4 μg and ~3.0 μg, respectively. POP was analyzed using the modified ash-hydrolysis method described previously with spectrophotometric detection at 885 nm^35,36^. The detection limit for POP is ~0.3 μg. It is important to note that measured particulate N and P are not devoid of inorganic N (e.g., aerosol-derived particulate nitrogen species) and P (e.g., polyphosphate granules), respectively. Furthermore, POM analyzed using this protocol includes contributions of dead materials in addition to live plankton cells, including a wide diversity of heterotrophs.

PCOD was quantified using the new, modified assay^7^ based on the determination of residual potassium dichromate following organic matter oxidation with silver sulfate as the catalyst under the strongly acidic condition at 150 °C for 2h^37–39^. As dichromate does not oxidize ammonium, the assay aims explicitly to quantify the oxygen demand from organic carbon (but not organic nitrogen). To remove the interference of chloride ions from the precipitation of silver chloride, mercuric sulfate was added^40^. Dichromate was quantified by absorbance at 600 nm using HACH-certified phthalate-based COD standards. We could not directly quantify the detection limit for PCOD as the PCOD chemistry method is highly sensitive (see Technical Validation).

## Data Records

Data of GO-POPCORN are publicly available in CSV format uploaded to Dryad for Version 1 (10.5061/dryad.d702p)^41^ and Version 2 (10.5061/dryad.05qfttf5h)^42^. GO-POPCORN datasets are distributed under a CC0 1.0 Universal Public Domain Dedication license.

## Technical Validation

In GO-POPCORN v1, most studies used similar techniques and sample volumes, but there are many slight deviations in the technical approach, including the measurement sensitivity, detection limits, the number of replicates, and the overall cleanliness (i.e., contamination) of procedures^9^. It is also worth noting that the POP measurements were grossly undersampled compared to POC and PON measurements in GO-POPCORN v1.

In GO-POPCORN v2, the POM samples were collected and quantified using consistent protocols. Before POM sampling, all the carboys used were rinsed at least twice with the pre-filtered underway seawater. The filtered volume of seawater was consistent between all POM (POC/N and POP) samples at each station and varied on a per-station basis to ensure that the amount of collected material was minimally impacted by the difference in filtration time. Initial rinsing and the large sampling volume were aimed at reducing the effect of a time delay caused by the underway system. The methods used for quantifying POC/N^43^ and POP^36^ are based on previously described and validated standard techniques.

POM described in this dataset are “small size-class” samples, where a 30 µm nylon mesh pre-filter was attached to the underway outlet to remove large plankton and particulates. In the Southern Ocean Section of the P18 cruise, we have separately collected “large-class” of POM >30 µm and showed that the larger particles constitute, on average, 17% of the total POC and PON concentrations and 31% of the total POP concentration^32^. The same study showed that a large size fraction of POM in P18 had statistically lower C:P, C:N, and N:P compared to a small size fraction of POM. However, the general effect of particle size on the C:N:P stoichiometry of POM is not yet clear.

For the technical validation of the novel PCOD assay, we tested for (1) interference using standard additions of a HACH-certified phthalate-based COD standard, (2) a linear correspondence between input amounts and absorbance, (3) the degree of variance with respect to POC measurement technique, and (4) biases for different substrates. In summary, we found that (1) the sample interference is limited, (2) there is indeed a linear relationship between filtered sample volume and PCOD, (3) variance for PCOD is higher compared to POC; hence it is vital to prepare and oxidize the high volume of POC to minimize relative error and ensure accurate determination of -O_2_:C, and (4) a high correspondence between theoretical and observed values for different substrates. A full detailed description of PCOD assay validation is described elsewhere^7^.

## Usage Notes

This dataset is the most comprehensive global compilation of surface POM and PCOD. By combining this dataset with datasets of temperature, nutrients, and plankton community composition, regional and global drivers of C:N:P:-O_2_ can be identified. The dataset is also useful for evaluating outputs from ocean biogeochemical models with flexible C:N:P:-O_2_ stoichiometry, with important implications for future ocean carbon, nitrogen, and oxygen dynamics.

## Data Availability

Code and data used to reproduce all the figures and tables are available in the GitHub repository https://github.com/tanio003/GOPOPCORN_Data_Codes and archived here (10.5281/zenodo.6967484)^44^.
